# The First Bacterial Endocarditis Due to *Achromobacter xylosoxidans* in a Dog

**DOI:** 10.3390/pathogens10121580

**Published:** 2021-12-03

**Authors:** Verena Steiner, Adriana Cabal Rosel, Werner Ruppitsch, Franz Allerberger, Alejandra Carranza Valencia, Mato Markovic, Nicole Luckschander-Zeller, Michael P. Szostak, Joachim Spergser, Igor Loncaric, Frank Künzel

**Affiliations:** 1 Department for Companion Animals and Horses, Clinical Unit of Internal Medic and Small Animals, University of Veterinary Medicine, 1210 Vienna, Austria; Alejandra.CarranzaValencia@vetmeduni.ac.at (A.C.V.); Mato.Markovic@vetmeduni.ac.at (M.M.); Nicole.Luckschander@vetmeduni.ac.at (N.L.-Z.); frank.kuenzel@vetmeduni.ac.at (F.K.); 2 Institute of Medical Microbiology and Hygiene, Austrian Agency for Health and Food Safety, 1090 Vienna, Austria; adriana.cabal-rosel@ages.at (A.C.R.); werner.ruppitsch@ages.at (W.R.); franz.allerberger@ages.at (F.A.); 3 Institute of Microbiology, University of Veterinary Medicine, 1210 Vienna, Austria; Michael.Szostak@vetmeduni.ac.at (M.P.S.); joachim.spergser@vetmeduni.ac.at (J.S.); Igor.Loncaric@vetmeduni.ac.at (I.L.)

**Keywords:** newly emerging bacteria, bacteremia, reserve antibiotic, endocarditis, canine

## Abstract

Infectious endocarditis (IE) in dogs is often associated with a high mortality rate as diagnostic work-up as well as antibiotic treatment might be challenging. The present case describes bacteremia in a dog caused by *Achromobacter* *xylosoxidans,* leading to an infectious endocarditis. *Achromobacter xylosoxidans* (*A.* *xylosoxidans*) is an aerobic Gram-negative rod-shaped bacterium, which has been associated with multiple nosocomial opportunistic diseases in human medicine. One such manifestation of *A.* *xylosoxidans* infection is endocarditis. *A.* *xylosoxidans* infections are challenging to treat due to the reduced effectiveness of a wide range of antimicrobial agents. To date, only a few case reports of infections with *A.* *xylosoxidans* in animals have been described. This is the first case report of *A.* *xylosoxidans* endocarditis in a dog. Whole-genome sequencing was performed to determine the sequencing type and to gain more information about this bacterium regarding its intrinsic resistance genes. With this case report, we seek to increase awareness of *A. xylosoxidans* as an opportunistic nosocomial pathogen in dogs and to provide a short summary regarding the current state of general knowledge and known resistance patterns.

## 1. Introduction

*Achromobacter xylosoxidans* (*A. xylosoxidans*) is an aerobic Gram-negative rod-shaped bacterium, first isolated in 1971 from the ear discharge of a human patient [1]. Since then, *A. xylosoxidans* has been associated with multiple opportunistic diseases in human medicine [2,3,4,5]. One of the manifestations of *A. xylosoxidans* infection is endocarditis [2].

To date, only a few case reports of infections with *A. xylosoxidans* in animals have been described [6,7,8,9]. This is the first case report of *A. xylosoxidans* endocarditis in a dog, to the best of our knowledge. This manuscript introduces *A. xylosoxidans* as a possible opportunistic nosocomial pathogen in dogs suffering from infectious endocarditis in veterinary medicine. Therefore, we give a short overview of *A. xylosoxidans* in general and its known resistance pattern. Furthermore, this manuscript discusses the important questions of possible infection routes and the risk of zoo–anthropogenic transmission.

## 2. Case Description

A large, spayed, 12-year-old, mixed-breed female dog was referred to the university clinic due to intermittent lameness, fever, and a newly detected heart murmur. According to past medical history, the dog had been repeatedly infested with ticks within the previous year. In addition, the dog had lost weight over the last 3 months after suffering an episode of diarrhea, which was treated with an intravenous infusion at a private veterinary practice.

During the initial physical examination, the dog showed tachycardia (180 bpm), a bounding pulse, and a body temperature of 39.9 °C. A systolic and diastolic heart murmur grade 4/6 audible on the left chest side was identified on auscultation. The orthopedic examination revealed an alternating lameness between all four extremities and a painful palpation of the right hip.

Hematology and blood biochemistry revealed mild, non-regenerative, normocytic, and normochromic anemia (PCV 0.34 L/L; 0.37–0.55 L/L); mild leukocytosis (16.9 × 10^9^/L; 6–15 × 10^9^/L) with neutrophilia without left shift (13.6 × 10^9^/L; 3.3–11.25 × 10^9^/L); monocytosis (1.3 × 10^9^/L; <0.5 × 10^9^/L); moderate hypoalbuminemia (20 g/L; 25.8–47.3 g/L); severely increased C-reactive protein (205 mg/L; <35.0 mg/L); mildly increased troponin I (0.4 ng/mL; <0.1 ng/mL); as well as severe hypocobalaminemia (144.61 pmol/L; 221–590 pmol/L). Due to the aforementioned tick exposure, tests for the detection of antibodies against *Anaplasma* spp., *Ehrlichia* spp., *Borrelia burgdorferi,* the antigen of *Dirofilaria immitis* (SNAP 4DX test, IDEXX Laboratories, Westbrook, ME, USA), as well as PCR for *Bartonella* spp. were performed. All of these proved negative. Urine analysis, including dipstick, sediment, bacterial culture, and urine protein/creatinine ratio, was unremarkable.

The thoracic radiographic imaging of the dog appeared normal. Radiographic examination of the hips showed bilateral dysplasia with pronounced right-sided coxarthrosis. The abdominal ultrasound gave evidence of a hyperechoic striated intestinal mucosa suspicious for chronic enteropathy, mildly enlarged intestinal lymph nodes, and an inhomogeneous hypoechogenic nodulous splenomegaly.

**Figure 1:**
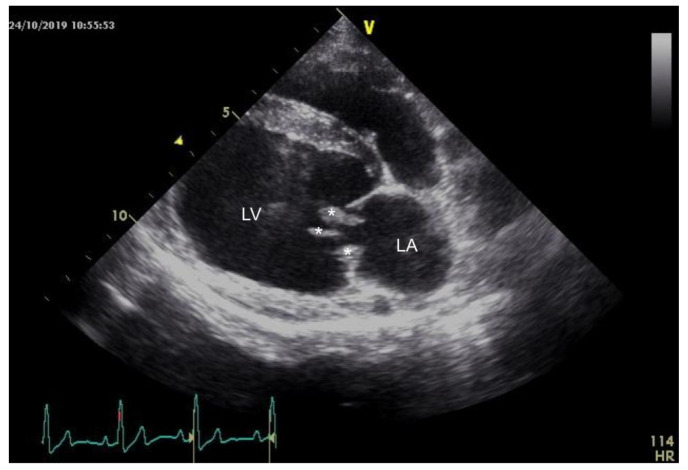
Right parasternal long axis of the left heart: Asterisk marks the irregular, highly mobile vegetative lesions of the mitral valve. LV = left ventricle; LA = left atrium.

The echocardiography revealed nodular, irregular, and highly mobile vegetative lesions of the mitral valve and a nodular lesion of the aortic valve (Figure 1), which led to a moderate mitral valve insufficiency and moderate to severe aortic valve insufficiency.

At the time of examination, a mild left ventricular volume overload (LVDd 48–50 mm, LVDdN 1.86–1.94) and a normal systolic function (FS 36.6–38%) were detected. Left atrial size was normal (La/Ao 1.3).

Based on the clinical signs and the results of the echocardiography, endocarditis with secondary polyarthritis was suspected. Subsequent diagnostics included blood culture, arthrocentesis of the carpal and tarsal joint followed by cytological and microbiological examinations, and a cytological examination of the spleen. Ten milliliters of blood was collected from the left jugular vein following shaving and disinfection with chlorhexidine and alcohol, which was then transferred aseptically into a blood culture bottle (Oxoid signal® Blood culture system BC0102M, Oxoid, Hampshire, UK). Afterward, the bottle was gently mixed and incubated at 37 °C.

An ultrasound-guided fine-needle aspiration of the spleen revealed splenitis with the predominance of small lymphocytes and neutrophils as the main cell type. Cytology of the synovial fluid revealed a pyogenic sterile arthritis. The microbiological examination, including mycoplasmas, was negative. Based on these results, secondary immune-mediated polyarthritis was suspected.

Due to the poor clinical condition of the dog, antibiotic treatment with amoxicillin-clavulanic acid (22 mg/kg TID IV) and marbofloxacin (4 mg/kg SID IV) was initiated as reported elsewhere for suspected IE with negative blood culture results [10]. A multi-modal analgesic regimen (buprenorphine 10 µg/kg QID IV, meloxicam initially with 0.2 mg/kg SID IV, then tapered to 0.1 mg/kg SID IV, pregabalin 2 mg/kg BID PO) was initiated as well. In addition, the hypocobalaminemia was treated by weekly supplementation with hydroxocobalamin 1000 µg/dog SC.

The dog showed a transient improvement in the clinical signs, but the fever continued despite antibiotic therapy. After seven days, the initial blood cultures tested positive for *A. xylosoxidans*, identified at the species level by matrix-assisted laser desorption/ionization-time of flight (MALDI-TOF) mass spectrometry (Bruker Daltonik, Heidelberg, Germany). According to the literature [11,12] and due to the lack of interpretative criteria for standard antimicrobial susceptibility tests in dogs, the antibiotic treatment was changed to meropenem (12 mg/kg BID IV). Twenty-four hours after initiating the treatment, fever resolved (38.7 °C), C-reactive protein level decreased (89 mg/L; <35.0 mg/L), and hematologic abnormalities returned to normal. A second blood culture was performed on the day of discharge (5 days after starting meropenem), which still tested positive for *A. xylosoxidans*. We recommended continuing the therapy with meropenem, however, the owner decided to discontinue antibiotic treatment due to financial and personal issues. The dog was euthanized a few days later as a result of clinical deterioration.

Whole-genome sequencing (WGS) was performed by isolating and sequencing bacterial DNA as previously described [13] to gain more insight into the detected *A. xylosoxidans* strain. De novo assembly of raw reads and WGS data analysis were performed as previously described [14,15]. Species identification was confirmed with the JSpecies workspace using the average nucleotide identity via Basic Local Alignment Search Tool (BLAST) (ANIb) analysis tool [16]. Classical multilocus sequence typing (MLST) data were extracted from WGS sequence data using *Achromobacter* MLST Databases hosted at PubMLST (https://pubmlst.org/achromobacter/, accessed on 25 September 2020) [17]. Based on the allelic profile of the seven loci, the PubMLST databases revealed that the present *A. xylosoxidans* strain belonged to the sequence type (ST) 201.

The identification of acquired resistance genes and chromosomal mutations was performed using the Comprehensive Antibiotic Resistance Database (CARD; https://card.mcmaster.ca/home, accessed on 26 May 2020) [18]. The *A. xylosoxidans* strain harbored genes for a narrow-spectrum class D-lactamase *bla*_OXA114a_ and the efflux pumps AxyXY-OprZ. The minimum inhibitory concentration (MIC) was determined by Etest (bioMérieux, Marcy l’Étoile, France) for imipenem, meropenem, and ceftazidime, according to the manufacturer’s recommendation, resulting in an MIC of 1 µg/mL for imipenem and meropenem, and 2 mg/L for ceftazidime.

## 3. Discussion

This is the first case describing IE in a dog with bacteremia due to *A. xylosoxidans*.

IE is an infrequently documented disease in dogs (prevalence (0.09–6.6%)) [10], and it is most often associated with a high mortality rate due to serious complications. However, IE in animals might be underdiagnosed, as diagnostic work-up is difficult. Thus, an adapted scheme of the modified Duke criteria is used as guidance for a reliable diagnosis of endocarditis in dogs and cats [10]. Based on the modified Duke criteria, the final diagnosis for IE in the present case was based on a positive echocardiogram (one major criterion) as well as on the positive blood culture and suspicious clinical signs (fever, newly emerged heart murmur, polyarthritis) in a medium- to large-sized dog (at least four minor criteria). The bacteria, commonly associated with IE in dogs, included Gram-positive cocci (51%) (*Staphylococcus* spp. (24%) and *Streptococcus* spp. (37%)), Gram-negative rods (22%), facultative Gram-positive rods (5%), *Actinomyces* spp. (2%), *Mycobacterium* spp. (2%), and *Bartonella* spp. (20%) [19]. IE associated with Gram-negative bacteria is more often detected in dogs [19], in comparison to humans where Gram-positive bacteria mainly induce IE. No reports of IE caused by *A. xylosoxidans* in dogs exist, even though it belongs to the Gram-negative class of bacteria.

According to the literature, this is the first case report of bacterial endocarditis due to *A. xylosoxidans* in dogs. *A. xylosoxidans* has been isolated from a wide range of clinical material [1,20] associated with multiple infectious diseases [2,3,4] in humans; most of the cases are opportunistic nosocomial infections [2,5,20,21]. Although the natural habitat of *A. xylosoxidans* is unknown, the mode of transmission in hospitals is considered to be through contaminated fluids used for diagnostics or therapy, or indwelling devices [5,20,21,22,23]. In particular, *A. xylosoxidans* endocarditis was mostly associated with catheter-related bacteremia in a human case series [2]. The few reported infections with *A. xylosoxidans* in animals have been mostly nosocomial infections following invasive interventions: infection of a total hip prosthesis in a dog [8], hemolytic anemia associated with endocarditis in rabbits after artificial creation of an aortic insufficiency [9], catheter-associated infections in baboons [6], and lower urinary tract infection in a cat with chronic kidney insufficiency [7]. Other infection routes for animals must be investigated; cross-kingdom transmission between plants, fungi, and animals was confirmed [24]. Furthermore, *A. xylosoxidans* was detected in the intestine of ticks (*Ixodes* spp.) as part of the unspecific bacterial flora [25]. Whether transmission by ticks while feeding or contact with contaminated soil could be a possibility has to be further investigated. However, based on this information and the clinical history of our dog, different routes of infection, e.g., the previous intravenous infusion therapy or repeated tick infestations, could be possible, but are speculative because of the retrospective nature of the case report.

Regarding the genetic diversity of the genus *Achromobacter*, 485 different sequence types (ST) have been reported according to public records (https://pubmlst.org/organisms/achromobacter-spp, accessed on 15 October 2020). The detected ST201 in our case was first reported in a human patient suffering from cystic fibrosis (CF) [4]. In general, *Achromobacter* spp. have emerged in CF patients, with *A. xylosoxidans* showing the highest prevalence (7.4–10.4%) [26] and mainly establishing a form of chronic colonization in CF patients (>80%) [26]. *A. xylosoxidans* was also detected in the domestic and outdoor environment of patients with CF [27]. To the best of our knowledge, the reported dog did not have contact with any human patients suffering from cystic fibrosis. Even though an indirect transmission of *Achromobacter* spp. between cystic fibrosis patients was observed [28], it requires further research to evaluate whether animals such as dogs could be a risk factor for zoo–anthropogenic transmission of these particular pathogens.

*A. xylosoxidans* infections are challenging to treat due to the reduced effectiveness of a wide range of antimicrobial agents [1,2,20,29]. *A. xylosoxidans,* as a non-fermentative Gram-negative bacterium, is intrinsically resistant to benzylpenicillin, first- and second-generation cephalosporins, glycopeptides, lipoglycopeptides, fusidic acid, macrolides, lincosamides, streptogramins, rifampicin, and oxazolidinones [30]. Based on the European Committee on Antimicrobial Susceptibility Testing (EUCAST), ampicillin, amoxicillin, ceftriaxone, cefotaxime, and ertapenem are additionally ineffective against *A. xylosoxidans* [30]. In total, 50 conserved drug-resistance-associated genes have been described in different isolates of *A. xylosoxidans* [29]. In general, intrinsic resistance of *A. xylosoxidans* is commonly associated with two efflux pumps, AxyABM and AxyXY-OprZ, and a narrow-spectrum class D β-lactamase OXA-114 [29]. The *A. xylosoxidans* strain of the present case harbored genes for the narrow-spectrum class D β-lactamase OXA-114 and the efflux pump AxyXY-OprZ. The efflux pump AxyXY-OprZ was associated with higher minimum inhibitory concentration (MIC) values of aminoglycosides, cefepime, carbapenems, some fluoroquinolones, tetracyclines, and erythromycin, while the class D β-lactamase OXA-114 showed efficient hydrolysis of piperacillin and, to a lesser extent, ticarcillin [29]. This efflux pump could eventually explain why the first antibiotic treatment choice (marbofloxacin) was not effective enough in our case. Currently, a novel β-lactamase with carbapenemase activity in a meropenem-resistant clinical isolate was documented [31]. Due to the positive treatment response reported elsewhere [10], carbapenems and later-generation cephalosporins are often used in *A. xylosoxidans* infections. However, newly developing resistance genes, as aforementioned [31], may reduce the effectivity of these antibiotics. However, clinical isolates often express resistance genes phenotypically different from their genotype [29]; therefore, susceptibility testing is essential in this patient.

The choice of an appropriate antibiotic should be based on microbiological criteria, such as the minimum inhibitory concentration (MIC), and pharmacological criteria, including the achievable concentration of the antibiotic at the clinical site as well as clinical efficacy. All these parameters take clinical breakpoints into account: clinical breakpoints are the concentrations of antibiotics used to define whether infection by a particular bacterial strain/isolate is likely to be treatable in a patient [32].

Because of the missing clinical breakpoints for *A. xylosoxidans*, a semi-quantitative susceptibility test such as the agar disk diffusion test could not be performed in the present case. To obtain MICs, an Epsilometer test (Etest) was performed with three antibiotics (imipenem, meropenem, and ceftazidime) based on the aforementioned intrinsic resistance pattern for *A. xylosoxidans*. Due to its known effectiveness against *A. xylosoxidans* infections in humans and its superior bactericidal activity compared to cephalosporins [11,33], meropenem was chosen for the antibiotic treatment in the present case. In addition, it can be administered as an IV bolus in small fluid volumes and shows fewer side effects than imipenem [33]. The used dosage (12 mg/kg BID IV) was chosen based on the published treatment recommendations for dogs [34]. Due to the lack of reported clinical breakpoints for *A. xylosoxidans* in dogs, it is unknown whether a serum concentration of meropenem was achieved to inhibit *A. xylosoxidans* in our case. However, one pharmacokinetic study reported that bacteria having an MIC of 0.12 µg/mL could be treated with dosages as low as 8 mg/kg, SC every 12 h in infections of the soft tissues or the urinary tract [35]. Nevertheless, higher dosages (12 mg/kg every 8 hours SC) are recommended to eliminate more resistant bacteria with an MIC of 1 µg/mL [35]. Based on this study, a higher dosage of meropenem than 12 mg/kg BID IV may have been necessary to achieve an appropriate serum concentration in our case, as the Etest resulted in an MIC of 1 µg/mL for meropenem. In contrast to the aforementioned data, our dog received the antibiotic IV, which lead to a higher serum concentration. However, in our case, the dog received the antibiotic for only several days due to the owner’s decision. As known from other more resistant bacteria, antibiotic treatment is often necessary for several weeks [10]; so, treatment time for our case was not long enough. Specific treatment recommendations for *A. xylosoxidans,* especially for endocarditis, need to be developed, as all effective antibiotics such as meropenem belong to the critically important antibiotic substance in human medicine [36]. Carbapenems have no approved use in veterinary medicine but can occasionally be applied in patients with bacterial-resistant infections. Because there have been reports of meropenem-resistant clinical isolates, a susceptibility test is essential to confirm the chosen antibiotic’s effectiveness. Nevertheless, further studies are required with regards to clinical breakpoints and in vivo MICs of different antibiotics for *A. xylosoxidans.*

## 4. Conclusions

In conclusion, our case report highlights the ST 201 of *A. xylosoxidans* as a potential pathogen causing infectious endocarditis in dogs. Further investigations are needed to confirm possible transmission routes in animals and to determine the risk of zoo–anthropogenic transmission. Known intrinsic resistance and current in vitro susceptibility tests must be considered for adequate antibiotic treatment, especially if they are refractory to standard antibiotic treatment regimens. In vivo MICs and clinical breakpoints should be established for bacteria such as *A. xylosoxidans* in order to select adequate antibiotics with appropriate effects in target tissues.
